# Utilizing Spontaneous Ventilation Modes in Patients Underwent Corrective Surgery for Right Ventricular Outflow Tract Obstructive Congenital Heart Disease: A Crossover Study

**DOI:** 10.31083/j.rcm2405143

**Published:** 2023-05-11

**Authors:** Xiaolei Gong, Limin Zhu, Mingjie Zhang, Yujie Liu, Chunxiang Li, Zhuoming Xu, Jinghao Zheng

**Affiliations:** ^1^Cardiac Intensive Care Unit, Department of Thoracic and Cardiovascular Surgery, Shanghai Children’s Medical Center, Shanghai Jiao Tong University School of Medicine, 200127 Shanghai, China; ^2^Department of Thoracic and Cardiovascular Surgery, Shanghai Children’s Medical Center, Shanghai Jiao Tong University School of Medicine, 200127 Shanghai, China

**Keywords:** congenital heart disease, mechanical ventilation, cardiac-pulmonary interaction, right ventricle outflow tract obstruction, spontaneous ventilation modes

## Abstract

**Background:**

This study aimed to determine whether the hemodynamics of patients with right ventricle outflow tract obstructive congenital heart disease (RVOTO-CHD) improve after corrective surgery by changing the ventilation mode.

**Methods:**

Patients with RVOTO-CHD who underwent corrective surgery were enrolled in this study. Echocardiography and advanced hemodynamic monitoring were performed using the pulse indicator continuous cardiac output (PiCCO) technology in the pressure-regulated volume control (PRVC) mode, followed with switching to the pressure support ventilation (PSV) mode and neurally adjusted ventilatory assist (NAVA) mode in random order.

**Results:**

Overall, 31 patients were enrolled in this study from April 2021 to October 2021. Notably, changing the ventilation mode from PRVC to a spontaneous mode (PSV or NAVA) led to better cardiac function outcomes, including right ventricular cardiac index (PRVC: 3.19 ± 1.07 L/min/$m^{2}$ *vs.* PSV: 3.45 ± 1.32 L/min/$m^{2}$ *vs.* NAVA: 3.82 ± 1.03 L/min/$m^{2}$, *p *< 0.05) and right ventricle contractility (tricuspid annular peak systolic velocity) (PRVC: 6.58 ± 1.40 cm/s *vs.* PSV: 7.03 ± 1.33 cm/s *vs.* NAVA: 7.94 ± 1.50 cm/s, *p *< 0.05), as detected via echocardiography. Moreover, in the NAVA mode, PiCCO-derived cardiac index (PRVC: 2.92 ± 0.54 L/min/$m^{2}$ *vs.* PSV: 3.04 ± 0.56 L/min/$m^{2}$ *vs.* NAVA: 3.20 ± 0.62 L/min/$m^{2}$, *p *< 0.05), stroke volume index (PRVC: 20.38 ± 3.97 mL/$m^{2}$ *vs.* PSV: 21.23 ± 4.33 mL/$m^{2}$ *vs.* NAVA: 22.00 ± 4.33 mL/$m^{2}$, *p *< 0.05), and global end diastolic index (PRVC: 295.74 ± 78.39 mL/$m^{2}$ *vs.* PSV: 307.26 ± 91.18 mL/$m^{2}$ *vs.* NAVA: 323.74 ± 102.87 mL/$m^{2}$, *p *< 0.05) improved, whereas extravascular lung water index significantly reduced (PRVC: 16.42 ± 7.90 mL/kg *vs.* PSV: 15.42 ± 5.50 mL/kg *vs.* NAVA: 14.4 ± 4.19 mL/kg, *p *< 0.05). Furthermore, peak inspiratory pressure, mean airway pressure, driving pressure, and compliance of the respiratory system improved in the NAVA mode. No deaths were reported in this study.

**Conclusions:**

We found that utilizing spontaneous ventilator modes, especially the NAVA mode, after corrective surgery in patients with RVOTO-CHD may improve their right heart hemodynamics and respiratory mechanics. However, further randomized controlled trials are required to verify the advantages of spontaneous ventilation modes in such patients.

**Clinical Trial Registration:**

NCT04825054.

## 1. Introduction

Patients with right ventricle outflow tract obstructive congenital heart diseases (RVOTO-CHDs), such as tetralogy of Fallot (TOF), pulmonary atresia with ventricular septal defect (PA/VSD), and TOF-type double outlet of the right ventricle with subaortic ventricular septal defect (TOF-type DORV) may develop systolic and/or diastolic right ventricular dysfunction postoperatively due to right ventriculotomy [1, 2]. In such patients, conventional positive pressure ventilation may restrict venous return, as well as increase the afterload of the impaired right ventricle and the severity of pulmonary valve regurgitation, leading to reduce right ventricular performance and left ventricular preload, further causing low cardiac output syndrome [3, 4, 5].

In the 1990s, children with TOF after corrective surgery were thought to have restrictive right ventricular physiology [6], but negative pressure ventilation by improving blood flow in the pulmonary circulation was possibly beneficial for them [7, 8]. However, in current clinical practice, it is difficult to achieve negative pressure ventilation in young infants. Remarkably, the mode of spontaneous ventilation has been found to be more similar to negative pressure ventilation approach. This inspired us to think about whether the use of spontaneous breathing mode could improve the postoperative hemodynamics of children with RVOTO-CHD. Notably, pressure support ventilation (PSV) is the most commonly used spontaneous ventilation mode, whereas neurally adjusted ventilatory assist (NAVA)—a newer spontaneous mode invented in the 1990s—collects diaphragmatic electromyographic signals from children via an electrical activity of diaphragm (EAdi) catheter and proportionally amplifies the pressure support amplitude [9]. Berger *et al*. [10] reported that compared with the PSV mode, when NAVA is modified according to the breathing pattern, right ventricular performance is less impaired in patients with cardiac impairment. This study aimed to determine whether the hemodynamics of patients with RVOTO-CHD improve after corrective surgery by changing the ventilation mode.

## 2. Methods

This study was conducted in accordance with the Declaration of Helsinki (as revised in 2013) and approved by the Institutional Health Research Ethics Board of Shanghai Children’s Medical Center, Shanghai Jiao Tong University School of Medicine (number: SCMC-CHC2021006). Informed consent was obtained from guardians of the patients for participation in this study. Notably, this study is registered with ClinicalTrials.gov (registration number: NCT04825054).

### 2.1 Setting

This single-center prospective crossover study was conducted in patients with RVOTO-CHD admitted to the cardiac intensive care unit of Shanghai Children’s Medical Center. Patients who underwent corrective surgery for RVOTO-CHD from April 2021 to October 2021 were enrolled in this study. The standard corrective procedure for RVOTO-CHD involved the following steps: resection of the obstructive site, enlargement of the right ventricular outflow tract (RVOT) using transannular patch repair, and closure of the intracardiac shunt (e.g., ventricular septal defect).

### 2.2 Patient Selection

Patients who met the following criteria after open-heart surgery were enrolled in this study: (1) diagnosed with RVOTO-CHD, including TOF, PA/VSD, TOF-type DORV, or pulmonary stenosis; (2) underwent corrective surgery; (3) spontaneous recovery of breathing; and (4) no excessive blood loss (chest drainage ≤3 mL/kg/hr within 6 hours). The exclusion criteria were as follows: patients with (1) arrhythmias, (2) residual cardiac defects (e.g., residual VSD or RVOTO), (3) severely low cardiac output (CI <1.8 L/min/$m^{2}$), (4) diaphragm paralysis, (5) pneumothorax or pleural effusion, and (6) no EAdi signal within the first 4 hours after insertion of the EAdi catheter.

### 2.3 Study Protocol

All patients were sedated with sufentanil and midazolam and ventilated using the pressure-regulated volume control (PRVC) mode of a Servo-i ventilator (v.7.00.04, Maquet Critical Care, Solna, Sweden). The baseline ventilator parameters were set as follows: tidal volume (Vt), 8–10 mL/kg; respiratory rate, 20–30 breaths/minutes; ${\mathrm{FiO}}_{2}$, 40%–50%; and positive end-expiratory pressure (PEEP), 4–5 ${\mathrm{cmH}}_{2}$O. For cardiac output monitoring, a 3F thermodilution catheter (PV2013L07-A, Pulsion Medical System, Feldkirchen, Germany) was percutaneously inserted into the left or right femoral artery under ultrasound guidance. The evaluation was started using the PRVC mode for 30 min after insertion and confirming the correct positioning of the EAdi catheter (8 Fr/100 cm, Maquet Critical Care, Solna, Sweden) via a specific function of the ventilator (EAdi catheter positioning) at 12–24 hours after the surgery when the patients’ spontaneous breath recovered. The children then underwent the PSV mode (PS, 6–8 ${\mathrm{cmH}}_{2}$O; ${\mathrm{FiO}}_{2}$, 40%–50%; PEEP, 4 ${\mathrm{cmH}}_{2}$O) and NAVA mode (NAVA level, 1.0–1.3 ${\mathrm{cmH}}_{2}$O/uV; ${\mathrm{FiO}}_{2}$, 40%–50%; PEEP, 4 ${\mathrm{cmH}}_{2}$O) ventilation for 30 minutes in random order. Notably, other ventilator settings (e.g., PEEP and ${\mathrm{FiO}}_{2}$) were kept constant throughout the study. Moreover, a previous study reported that the duration of 30 min is sufficient to observe hemodynamic changes [11].

At the end of ventilation using each mode for 30 minutes, echocardiography was performed to measure cardiac parameters, followed by thermodilution calibration with cold saline (5 mL, 4 °C for 3 times) and blood sample collection for the evaluation of gas analysis and NT-proBNP.

### 2.4 Hemodynamic and Gas Exchange Measurements

Hemodynamic parameters, including heart rate (HR), central venous pressure (CVP), mean arterial blood pressure (ABPm), cardiac index (CI) with thermodilution (CI-PiCCO, pulse indicator continuous cardiac output), stroke volume index (SVI), global end diastolic volume index (GEDI), global ejection fraction (GEF), left ventricle myocardial contractility index (DPmx), and extravascular lung water index (ELWI), were assessed using the second generation of Pulse indicator continuous cardiac output monitoring system (${\mathrm{PiCCO}}_{2}$) (v.3.1.0.8A, Pulsion Medical Systems, Feldkirchen, Germany). Moreover, cardiac function parameters, including RVOT diameter (RVOTD), velocity-time integral (VTI), right ventricular CI by echocardiography (RV-CI), a variation of inferior vena cava (IVCv; IVCv = [IVC diameter during expiration – IVC diameter during inspiration]/IVC diameter during expiration), tricuspid annular plane systolic excursion (TAPSE), tricuspid annular peak systolic velocity (S’), left ventricular diastolic diameter (LVDD), left ventricular systolic diameter (LVDS), left ventricular ejection fraction (LVEF), and the ratio of early diastolic mitral inflow to average mitral annular tissue velocity (E/e’), were determined using Vivid-E95 (GE Vingmed Ultrasound, Horten, Norway). In addition, blood gas parameters, including pH, ${\mathrm{PaCO}}_{2}$, ${\mathrm{PaO}}_{2}$, ${\mathrm{SaO}}_{2}$, Base excess (BE), ${\mathrm{PcvCO}}_{2}$, ${\mathrm{ScvO}}_{2}$, and lactate, were evaluated using ABL 850 (Radiometer, Copenhagen, Denmark). Further, N-terminal pro-B-type natriuretic peptide (NT-proBNP) was analyzed using TZ-320 (ReLIA, Wuxi, China). Parameters of respiratory mechanics, including respiratory rate, peak airway pressure (PIP), mean airway pressure (MAP), time of inspiration (Ti), tidal volume (Vt), dynamic driving pressure (DP), compliance of the respiratory system (Crs), peak EAdi (Edi-p), minimal EAdi (Edi-m), and inhaled fraction of oxygen (${\mathrm{FiO}}_{2}$), were recorded from the ventilator.

### 2.5 Data Collection

Baseline demographic data (including age, sex, height, and weight), details of the diagnosis, and information of intraoperative characteristics (such as cardiopulmonary bypass time, aortic clamp time, and clinical outcome) were collected.

### 2.6 Statistical Analysis

The SPSS v. 21.0 statistical software (IBM Corporation, Armonk, NY, USA) was used for statistical analyses. All statistical data were assessed using two-sided tests, and type I errors were set at 0.05. Continuous data with normal distributions are presented as mean ± standard deviation, and the differences between ventilation modes were compared using paired *t*-tests. Data that were not normally distributed are presented as median with interquartile range (IQR, 25th to 75th percentile), and differences between ventilation modes were compared using the Wilcoxon signed rank test.

## 3. Results

**Fig. 1.**
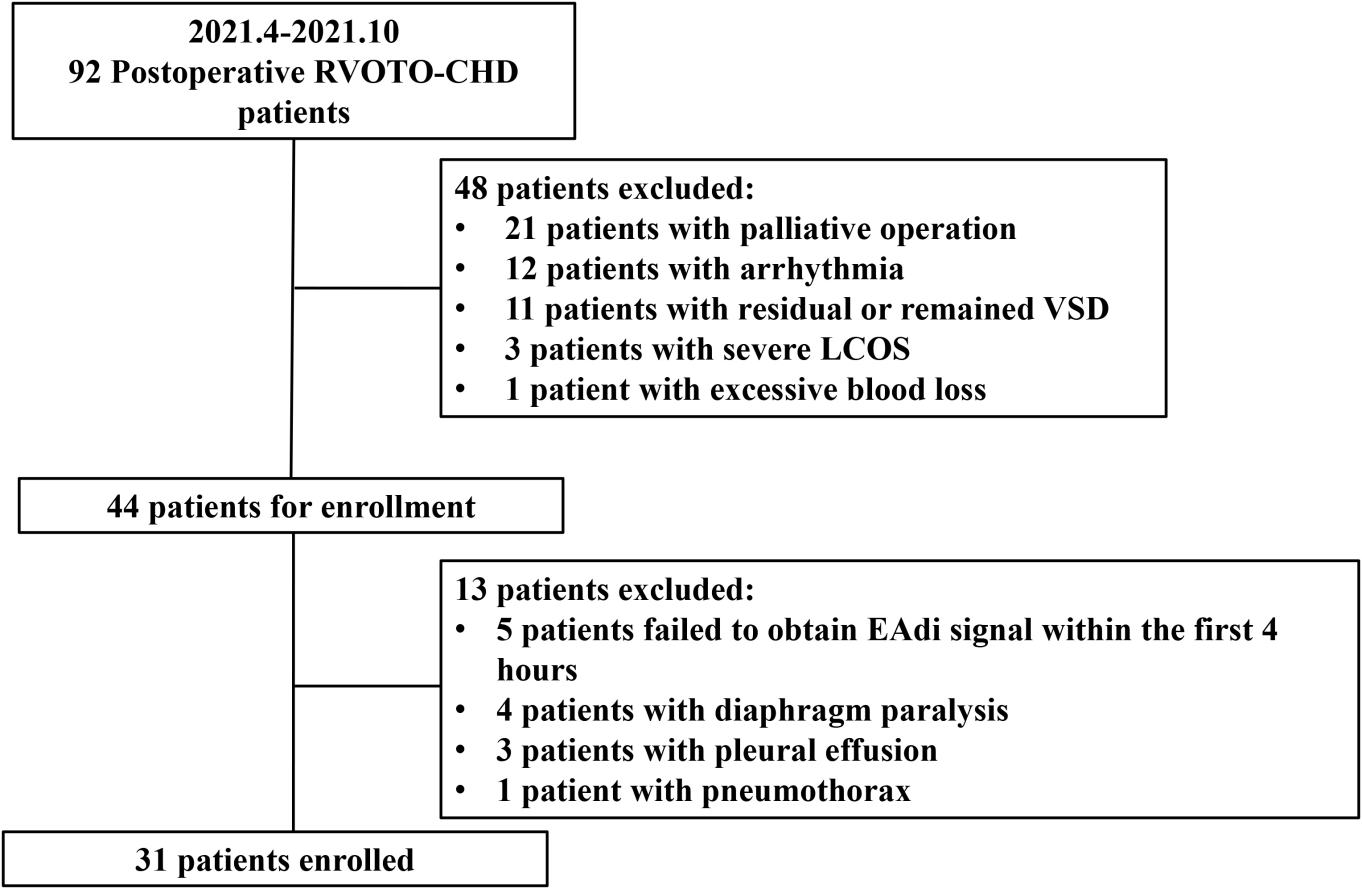
**Patient flowchart**. RVOTO-CHD, right ventricle outflow tract obstructive congenital heart disease; VSD, ventricular septal defect; LCOS, low cardiac output syndrome; EAdi, electrical activity of diaphragm.

From April 2021 to October 2021, 92 patients with postoperative RVOTO-CHD, including 53 males, with a mean age of 5 (4–7) months, were admitted to the cardiac care unit (patient flowchart is shown in Fig. 1). Of these 92 patients, 21 underwent palliative surgery, 12 developed arrhythmias before enrollment, 11 had residual or remained VSD due to high right ventricular pressure, 3 developed severe low cardiac output syndrome (CI <1.8 L/min/$m^{2}$) despite the optimization of volume management, and 1 experienced excessive blood loss within 6 hours. Of the remaining 44 patients, 3 with pleural effusion, 5 failed to obtain EAdi signal within the first 4 hours after the insertion of the EAdi catheter, 4 with diaphragmatic paralysis, and 1 with pneumothorax were excluded. Finally, 31 patients were included in this study.

### 3.1 Patient Characteristics and Perioperative Outcomes

Patient characteristics and perioperative outcomes of the enrolled patients are presented in Table 1. Remarkably, the median age of the enrolled patients was 5 months. Most patients were diagnosed with TOF (83.87%) and underwent corrective surgery. The median cardiopulmonary bypass time was 85 minutes, whereas the mean aortic clamp time was 48 minutes. In addition, transannular patch repair was performed in all patients during the reconstruction of the RVOT. Notably, only one patient required noninvasive ventilation support after weaning from the invasive ventilator because of dyspnea caused by laryngeal edema. No deaths were reported in this study.

**Table 1.** **General information and perioperative characteristics of patients**.

Variables (n = 31)	Value
Gender Male, n (%)	18 (58.6)
Age, months	5 (4, 7)
Height, cm	66.27 ± 1.02
Weight, kg	7.27 ± 0.28
BSA, $m^{2}$	0.36 ± 0.05
Diagnosis, n (%)	
∙ TOF	26 (84)
∙ PA/VSD	2 (6)
∙ DORV/PS (TOF type)	3 (10)
Cardiopulmonary Bypass time, min	85 (64, 100)
Aortic clamping time, min	48 (40, 58)
Invasive ventilation time, hours	48.2 (43.4, 65.5)
ICU LOS, d	5 (4, 5)
In-hospital LOS, d	13 (12, 17)
P-SOFA score	4.32 ± 0.45
NIV, n (%)	1 (3.23)
Mortality, n (%)	0 (0)

*Note*. Continuous data are presented as mean (SD) or median (IQR). Categorical data are presented as numbers (%). BSA, body surface area; DORV/PS, double outlet of right ventricle with subaortic ventricular septal defect and pulmonary stenosis; LOS, length of stay; NIV, noninvasive ventilation; P-SOFA, pediatric sequential organ failure assess; PA/VSD, pulmonary atresia with ventricular septal defect; TOF, tetralogy of Fallot.

### 3.2 Cardiac Function Measured via Echocardiography during Different Ventilation Modes

Cardiac function parameters measured via echocardiography are shown in Table 2. Compared with right ventricle (RV) hemodynamics during the use of the other two modes, the hemodynamics during the use of the NAVA mode was significantly improved. RV cardiac output improved by 10.72% and 19.75% during the use of NAVA mode compared with cardiac output during the PSV and PRVC modes, respectively. Notably, S’—a measurement of RV systolic function—also improved during the use of the NAVA mode compared with that in the other two modes (7.94 ± 1.50 *vs.* 7.03 ± 1.33 for PSV and 6.58 ± 1.40 for PRVC).

**Table 2.** **Cardiac function measured via echocardiography during different ventilation modes**.

	PRVC	PSV	NAVA	*p* value		
				PRVC *vs.* PSV	PSV *vs.* NAVA	PRVC *vs.* NAVA
RVOTD, mm	10.04 ± 1.88	10.04 ± 1.72	10.30 ± 1.88	0.98	0.07	0.100
RV-VTI, cm	9.46 ± 1.31	10.32 ± 1.50	11.68 ± 2.26	0.001	0.000	0.000
RV-CI, L/min/$m^{2}$	3.19 ± 1.07	3.45 ± 1.32	3.82 ± 1.03	0.003	0.564	0.035
IVCi, cm	0.60 ± 0.18	0.54 ± 0.17	0.55 ± 0.17	0.003	0.564	0.035
IVCe, cm	0.77 ± 0.16	0.74 ± 0.15	0.75 ± 0.16	0.123	0.543	0.444
IVCv	0.22 ± 0.17	0.27 ±.016	0.27 ± 0.14	0.036	0.98	0.067
S’, cm/s	6.58 ± 1.40	7.03 ± 1.33	7.94 ± 1.50	0.003	0.000	0.000
TAPSE, cm	0.56 (0.46, 0.64)	0.57 (0.51, 0.71)	0.64 (0.54, 0.78)	0.303	0.019	0.000
LVDD, cm	2.06 ± 0.35	2.07 ± 0.33	2.11 ± 0.35	0.924	0.317	0.401
LVDS, cm	1.37 ± 0.33	1.32 ± 0.31	1.33 ± 0.29	0.341	0.745	0.537
LVEF, %	66.15 ± 8.41	67.98 ± 7.83	67.97 ± 7.12	0.167	0.985	0.225
E/e’	11.71 (9.86, 13.83)	11.9 (9.63, 14.00)	11.63 (9.08, 12.88)	0.959	0.179	0.357

*Note*. Continuous data are presented as mean (SD) or median (IQR). Categorical data are presented as numbers (%). E/e’, the ratio of early diastolic mitral inflow to average mitral annular tissue velocity; IVCe, diameter of inferior vena cava during expiration phase; IVCi, diameter of inferior vena cava during inspiration phase; IVCv, variation of inferior vena cava; LVDD, left ventricle diastolic diameter; LVDS, left ventricle systolic diameter; LVEF, left ventricle ejection fraction; RV-CI, cardiac index of right ventricle; RV-VTI, velocity-time integral of right ventricle; RVOTD, diameter of right ventricular outflow tract; S’, tricuspid annular peak systolic velocity; TAPSE, tricuspid annular plane systolic excursion.

### 3.3 Basic Hemodynamics, NT-proBNP and PiCCO Monitoring during Different Ventilation Modes

Patients had higher systolic blood pressure during the use of NAVA mode (101.61 ± 12.78 mmHg) than during the use of the PRVC mode (96.35 ± 13.03 mmHg); however, the diastolic blood pressure and ABPm were not significantly different. Notably, CI and SVI measured using the thermodilution method tended to be higher with the use of the NAVA mode than with the use of the PRVC and PSV modes. Moreover, NT-proBNP was significantly lower in the NAVA mode than in the PSV and PRVC modes, as described in Table 3.

**Table 3.** **Basic hemodynamics and PiCCO monitoring during different ventilation modes**.

	PRVC	PSV	NAVA	*p* value		
				PRVC *vs.* PSV	PSV *vs.* NAVA	PRVC *vs.* NAVA
HR, beats/minute	144.84 ± 12.25	145.48 ± 12.70	146.48 ± 13.00	0.714	0.403	0.318
ABPs, mmHg	96.35 ± 13.03	100.55 ± 13.34	101.61 ± 12.78	0.088	0.547	0.022
ABPd, mmHg	54.65 ± 7.73	56.74 ± 7.78	56.97 ± 7.78	0.164	0.869	0.244
ABPm, mmHg	71.00 ± 9.10	74.10 ± 9.81	74.65 ± 10.05	0.058	0.726	0.112
CVP, mmHg	11.65 ± 2.58	12.03 ± 2.29	12.03 ± 2.47	0.28	1.000	0.296
VIS	15 (13, 17.38)	15 (12.5, 15.88)	15 (13, 16.63)	0.285	0.655	0.317
CI-PiCCO, L/minute/$m^{2}$	2.92 ± 0.54	3.04 ± 0.56	3.20 ± 0.62	0.096	0.005	0.001
SVI, mL/$m^{2}$	20.38 ± 3.97	21.23 ± 4.33	22.00 ± 4.33	0.089	0.036	0.002
GEDI, mL/$m^{2}$	295.74 ± 78.39	307.26 ± 91.18	323.74 ± 102.87	0.225	0.043	0.026
SVRI, dyn⋅s⋅${\mathrm{cm}}^{-\,5}$⋅$m^{2}$	1654 (1496, 1900)	1538 (1412, 1841)	1458 (1325, 1777)	0.367	0.055	0.061
GEF, %	27.37 ± 4.21	28.07 ± 4.53	28.20 ± 4.90	0.120	0.775	0.104
DPmx, mmHg/s	833.5 ± 280.74	924.5 ± 301.35	913.53 ± 309.58	0.007	0.644	0.156
ELWI, mL/kg	16.42 ± 7.90	15.42 ± 5.50	14.4 ± 4.19	0.150	0.030	0.039
NT-proBNP, pg/mL	17,000 (9850, 27,500)	17,000 (6350, 25,500)	16,000 (7445, 22,000)	0.338	0.032	0.001

*Note*. Continuous data are presented as mean (SD) or median (IQR). Categorical data are presented as counts (%). ABPs, systolic arterial blood pressure; ABPd, diastolic arterial blood pressure; CI-PiCCO, cardiac index measured by PiCCO system; CVP, central venous pressure; DPmx, left ventricle myocardial contractility index; ELWI, extravascular lung water index; GEDI, global end diastolic volume index; GEF, global ejection fraction; HR, heart rate; SVI, stroke volume index; SVRI, systemic vascular resistance index; VIS, vasoactive inotropic score.

### 3.4 Respiratory Mechanics, Blood Gas Parameters, and Other Laboratory Findings

Table 4 shows the respiratory mechanics parameters, blood gas parameters, and NT-proBNP for the three ventilation modes. The respiratory parameters, such as PIP, MAP, DP, and OI, were the lowest during the use of NAVA mode, whereas Vt and Crs were the highest during the use of NAVA mode.

**Table 4.** **Respiratory mechanics, blood gas parameters, and other laboratory findings**.

	PRVC	PSV	NAVA	*p* value		
				PRVC *vs.* PSV	PSV *vs.* NAVA	PRVC *vs.* NAVA
Freq, breaths/min	26.5 (22.8, 30.5)	27 (24, 31.3)	26.5 (25, 31.3)	0.059	0.837	0.087
PIP, ${\mathrm{cmH}}_{2}$O	15 (13, 17)	13 (12,15.3)	12 (10, 12.3)	0.000	0.000	0.000
MAP, ${\mathrm{cmH}}_{2}$O	7.5 (6, 9)	7 (6, 8.3)	6 (5, 7)	0.003	0.000	0.000
PEEP, ${\mathrm{cmH}}_{2}$O	4.5 (4, 5)	4.5 (4, 5)	4.5 (4, 5)	1.000	1.000	1.000
Ti, s	0.66 (0.6, 0.7)	0.61 (0.49, 0.7)	0.76 (0.67, 0.83)	0.043	0.000	0.000
Vt, mL	59.1 ± 15.6	54.7 ± 13.7	53.6 ± 14.3	0.001	0.535	0.013
Vt, mL/kg	8.2 ± 1.6	7.6 ± 1.4	7.5 ± 1.7	0.002	0.595	0.011
DP, ${\mathrm{cmH}}_{2}$O	10.9 ± 3.1	9.2 ± 1.9	6.9 ± 1.8	0.000	0.000	0.000
Crs, mL/${\mathrm{cmH}}_{2}$O/kg	0.8 ± 0.2	0.9 ± 0.2	1.1 ± 0.4	0.012	0.000	0.000
EAdi-P, uV	3.4 (1.8, 6.2)	3.9 (2.6, 5.0)	4.6 (3.1, 5.1)	0.462	0.551	0.183
EAdi-m, uV	0.3 (0.2, 0.8)	0.4 (0.2, 0.7)	0.5 (0.3, 0.7)	0.502	0.387	0.592
${\mathrm{FiO}}_{2}$	0.4 (0.39, 0.43)	0.4 (0.39, 0.43)	0.4 (0.39, 0.43)	1.000	1.000	1.000
pH	7.43 ± 0.03	7.43 ± 0.04	7.43 ± 0.04	0.080	0.588	0.254
${\mathrm{PaCO}}_{2}$, mmHg	40.9 ± 5.0	39.9 ± 5.3	41.0 ± 5.5	0.349	0.075	0.786
${\mathrm{PaO}}_{2}$, mmHg	117.1 ± 31.8	114.9 ± 31.1	119.9 ± 28.4	0.597	0.156	0.579
${\mathrm{PaO}}_{2}$/${\mathrm{FiO}}_{2}$	288.3 ± 87.8	283.1 ± 89.3	292.8 ± 80.6	0.630	0.207	0.668
OI	2.6 (2.0, 3.8)	2.6 (1.9, 3.9)	2.1 (1.9, 2.7)	0.367	0.000	0.000
${\mathrm{SaO}}_{2}$, %	99 (97.7, 99.5)	98.4 (97.3, 99.0)	98.8 (97.9, 99.4)	0.629	0.130	0.327
BE	2.3 ± 2.8	2.3 ± 3.1	2.8 ± 2.8	0.890	0.114	0.152
${\mathrm{PcvCO}}_{2}$, mmHg	48.5 ± 3.9	48.4 ± 5.1	49 ± 4.6	0.971	0.249	0.367
${\mathrm{ScvO}}_{2}$, %	68.1 ± 8.4	68.7 ± 9.6	67.9 ± 7.5	0.688	0.473	0.864
${\mathrm{SaO}}_{2}$-${\mathrm{ScvO}}_{2}$, %	30.1 ± 9.0	29.4 ± 9.6	30.7 ± 7.8	0.639	0.222	0.585
${\mathrm{PcvCO}}_{2}$-${\mathrm{PaCO}}_{2}$, mmHg	7.6 ± 3.7	8.5 ± 4.4	7.9 ± 4.2	0.343	0.319	0.671
LAC, mmol/L	0.8 (0.7, 1.4)	0.8 (0.7, 1.0)	0.8 (0.6, 1.2)	0.036	0.260	0.280

*Note*. Continuous data are presented as mean (SD) or median (IQR). Categorical data are presented as counts (%). BE, base excess; Crs, compliance of respiratory system; DP, driving pressure; EAdi-m, minimal voltage of electrical activity of diaphragm; EAdi-p, peak voltage of electrical activity of diaphragm; ${\mathrm{FiO}}_{2}$, inhaled fraction of oxygen; Freq, breath frequency; LAC, lactate; MAP, mean airway pressure; OI, oxygen index; PEEP, positive end-expiratory pressure; PIP, peak inspiratory pressure; Ti, inspiration time; Vt, tidal volume.

## 4. Discussion

To the best of our knowledge, information on the cardiopulmonary interaction affected by the ventilation mode using different hemodynamic monitoring methods is insufficient in children with CHD. This study found that the use of spontaneous breathing mode, especially the NAVA mode, could increase pulmonary blood flow measured via echocardiography, and the benefit of the spontaneous breathing mode could also be seen using the thermodilution technique. In addition, the respiratory mechanics were found to be improved in the PSV and NAVA modes.

According to the results of echocardiography, after switching to PSV and NAVA mode, the variation rate of the inferior vena cava in children with reduced intrathoracic pressure and increased intraabdominal pressure caused by diaphragm movement during spontaneous breathing increased significantly, and the indices of right ventricular contractility, such as TAPSE and S’, improved. We found that RV-VTI and RV-CI increased, and this finding was similar to the results of a study by Becker *et al*. [10]. Furthermore, the use of PiCCO catheter with thermodilution technique and pulse waveform analysis technique revealed that GEDI increased with the increased venous return due to spontaneous respiration as well as the cardiac output. Liet *et al*. [12] reported that cardiac output increased in children with low cardiac output after congenital heart disease surgery during the use of NAVA mode for 30 minutes. In the present study, right ventricular cardiac output measured by ultrasound (RV-CI) differed from that measured by the thermodilution method (CI-PiCCO) owing to the overestimation of RV-CI caused by pulmonary insufficiency in children with right ventricular transannular patch enlargement or by the use of different methods, such as that reported by Wetterslev *et al*. [13].

After switching to a spontaneous breathing mode, especially the NAVA mode, some respiratory mechanics also changed significantly due to decreased intrathoracic pressure during inspiration, resulting in a decrease in PIP, MAP, and DP and an improvement in Crs. These results are consistent with our previous study in children with single ventricles [14] and many other studies [12, 15, 16, 17]. In some studies of the NAVA mode, children ventilated with the NAVA mode had lower airway pressures than children ventilated with the continuous positive airway pressure mode, suggesting that the NAVA mode used before weaning may be more appropriate than the other modes [18, 19].

Some children may develop weaning-induced pulmonary edema (WIPO) after weaning from mechanical ventilation, mainly due to increased venous return and consequent improvement in right ventricular output as well as a displacement of the intraventricular septum to the left ventricle after ventilator discontinuation [20]. Notably, an increase in NT-proBNP and ELWI after spontaneous breathing can be an important predictor of WIPO [21]. In our study, NT-proBNP, ELWI, and echocardiographic parameters representing left ventricular filling pressure (E/e’) were also measured after switching the mechanical ventilation modes. We found that these indices did not significantly increase in the spontaneous breathing mode. NT-proBNP was lower in the NAVA mode compared with PRVC and PSV, and this was possibly related to the decrease in right ventricular pressure. The decrease in ELWI and NT-proBNP in the NAVA ventilation mode suggests that these children are less likely to develop WIPO.

This study had several limitations. First, no control group was included, and this was only a self-controlled study. Thus, whether hemodynamics and respiratory mechanics changed after surgery in children who did not use the NAVA or PSV mode remains to be investigated. Second, right ventricular pressure data were not collected in this study. Most children had mild tricuspid regurgitation; therefore, echocardiography may underestimate the right ventricular pressure. The patients included in this study were children aged 4–7 months; thus, the placement of a floating pulmonary artery catheter was difficult. Therefore, no right ventricular pressure measurements were performed in this study.

## 5. Conclusions

Utilizing spontaneous ventilator modes, especially the NAVA mode, after corrective surgery in patients with RVOTO-CHD could improve their right heart hemodynamics and respiratory mechanics. Further randomized controlled trials should be conducted to verify the advantages of spontaneous ventilation modes in such patients.

## Data Availability

The datasets used and/or analyzed during the current study are available from the corresponding author on reasonable request.
